# Common Symptoms and a Rare Diagnosis: A Case of Duodenal Gastrointestinal Stromal Tumor Presenting as Gastrointestinal Bleeding

**DOI:** 10.7759/cureus.69814

**Published:** 2024-09-20

**Authors:** Mona Amin, Ahmed Nageeb, Shadi Abuhashem, Abobakr Saleh, Esraa Awad, Rana Raed

**Affiliations:** 1 Internal Medicine, Faculty of Medicine, Cairo University, Cairo, EGY; 2 Internal Medicine, Al-Quds University, Jerusalem, PSE; 3 Internal Medicine, Cairo University, Cairo, EGY; 4 Internal Medicine, Zagazig University, Zagazig, EGY

**Keywords:** acute gastrointestinal bleed, duodenal, duodenal gist, endoscopic ultrasound (eus), gastrointestinal stromal tumor (gist), melena, subepithelial lesion

## Abstract

Duodenal gastrointestinal stromal tumors (D-GISTs) are a rare subtype of GISTs, accounting for only 4% to 5% of all GIST cases. This case report details the presentation, diagnosis, and management of a 48-year-old female who presented with melena and anemia and was eventually diagnosed with a D-GIST. The tumor was identified through imaging studies, and histopathology performed after surgical resection revealed a submucosal neoplasm composed of spindle cells with extensive hemorrhage and necrosis. Given the tumor's rarity and its challenging presentation, which can mimic other conditions such as pancreatic masses, the case underscores the importance of considering D-GIST in differential diagnoses of duodenal or pancreatic lesions. Surgical resection remains the cornerstone of treatment, with adjuvant therapy considered in high-risk cases to prevent recurrence.

## Introduction

Gastrointestinal stromal tumors (GISTs) are the most common mesenchymal neoplasms, originating from the interstitial cells of Cajal within the gastrointestinal (GI) tract [1]. They can occur anywhere along the GI tract, but most commonly in the stomach and small intestine [2,3]. Duodenal GISTs (D-GISTs), however, are quite rare, with an estimated prevalence of 4% to 5% of all GIST cases. Accurate diagnosis is often reliant on advanced imaging modalities, including endoscopic ultrasound (EUS) and CT, which are critical for assessing tumor characteristics and planning treatment strategies [1]. This is usually followed by a tissue biopsy with immunohistochemical analysis to confirm the diagnosis, with approximately 95% of all GISTs being positive for KIT (CD117). 

This report presents the case of a 48-year-old woman with a history of peptic ulcer disease who experienced recurrent melena and symptomatic anemia. Initially thought to be a recurrence of her ulcer disease, further evaluation uncovered a D-GIST. This case highlights the pivotal role of comprehensive imaging in the management of these rare tumors.

## Case presentation

A 48-year-old housewife with a past medical history of no special habits of medical importance presented to the ER complaining of black tarry stool for a duration of three days, which was indicative of melena. This was her second attack of melena, the first having occurred two years ago after taking non-steroidal anti-inflammatory drugs (NSAIDs) for headaches and was attributed to peptic ulcer disease (PUD) confirmed by endoscopy, which was treated medically. She also reported an increased sensation of fatigue and exertional shortness of breath that started around a month prior to her presentation. No hematemesis, abdominal pain, weight loss, or changes in bowel habits were reported. Her surgical history includes an appendectomy in childhood and a hernioplasty 10 years ago.

On physical examination, the patient was tachycardic (Table 1). Pallor was noted, but the examination was otherwise unremarkable, with no abdominal tenderness, masses, or signs of liver disease. There was no significant lymphadenopathy or stigmata of chronic liver disease.

**Table 1:** Results of physical examination

Vital signs	Values
Heart rate	110 bpm
Blood pressure	100/70 mmHg
Respiratory rate	15 bpm
Temperature	37.1°C
Oxygen saturation	96%

Routine lab work showed severe normocytic anemia (Table 2). The anemia itself, being normocytic, was attributed to acute bleeding. This was supported by reticulocytosis.

**Table 2:** Laboratory work-up MCV: Mean corpuscular volume, Hct%: Hematocrit, AST: Aspartate aminotransferase, ALT: Alanine aminotransferase, CRP: C-reactive protein, ESR: Erythrocyte sedimentation rate, HBsAg: Hepatitis B surface antigen, HCV Ab: Hepatitis C virus antibody

Parameters	Values	Normal range
Hemoglobin	6.5 g/dL	12.0 - 16.0 g/dL (female)
MCV	81 fL	80 - 100 fL
Reticulocytes	10%	0.5% - 2.5%
RBC count	2.2 million/cc	4.2 - 5.4 million/μL (female)
Hct%	21%	36% - 48% (female)
Sodium	138 mmol/L	135 - 145 mmol/L
Potassium	3.6 mmol/L	3.5 - 5.1 mmol/L
Urea	36 mg/dL	7 - 20 mg/dL
Creatinine	0.54 mg/dL	0.5 - 1.1 mg/dL (female)
AST	24	10 - 40 U/L
ALT	21	7 - 56 U/L
Bilirubin, total	0.9	0.1 - 1.2 mg/dL
Bilirubin, direct	0.1	0.0 - 0.4 mg/dL
CRP	16 mg/L	< 10 mg/L
ESR	50	0 - 20 mm/hr (female)
HBsAg	negative	negative
HCV Ab	negative	negative

Our approach to this case, where one episode of melena caused severe, symptomatic anemia, was to first exclude upper GI bleeding. The patient was initially managed with blood transfusion; she received 2 units of packed RBCs (PRBC) that improved her hemoglobin (HB) level to 8.5 g/dl. Upper endoscopy revealed a very large, well-defined, localized lesion in the duodenum with a smooth surface and ulceration, indicative of a subepithelial lesion (SEL) for differential diagnosis. Due to active bleeding, biopsies were not taken during the endoscopy. Consequently, alternative imaging studies were conducted to evaluate the bleeding.

The EUS revealed a very large, well-defined, localized complex cystic lesion, originating from the muscularis propria of the third part of the duodenum, measuring 6.5 cm x 5.5 cm. The lesion showed exophytic growth, overlapping but appearing separate from the pancreatic uncinate process, superior mesenteric artery (SMA), and superior mesenteric vein (SMV). Large areas of cystic breakdown with significant arterial supply were noted, highly suggestive of a GIST. A fine needle aspiration (FNA) biopsy could not be taken due to concerns of bleeding.

**Figure 1:**
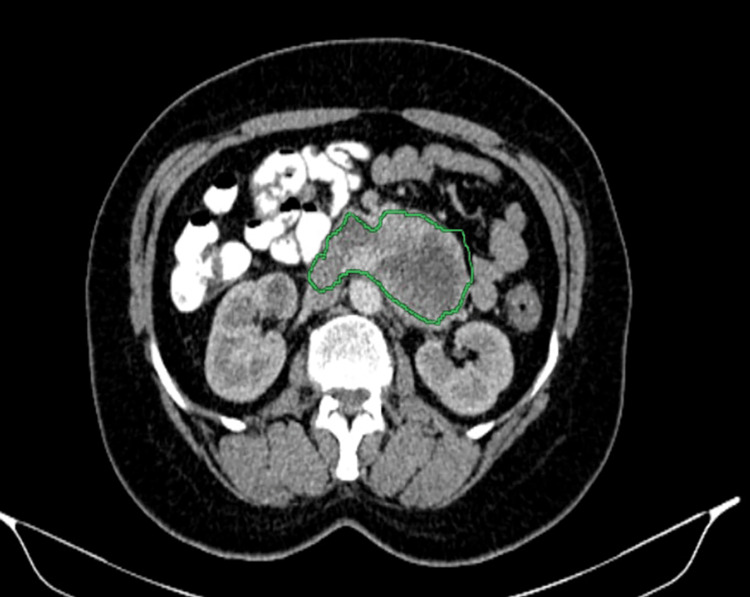
The axial CT scan of the abdomen shows a well-defined mass near the pancreas (outlined in green). The mass was diagnosed as a GIST involving the duodenum. GIST: Gastrointestinal stromal tumor

**Figure 2:**
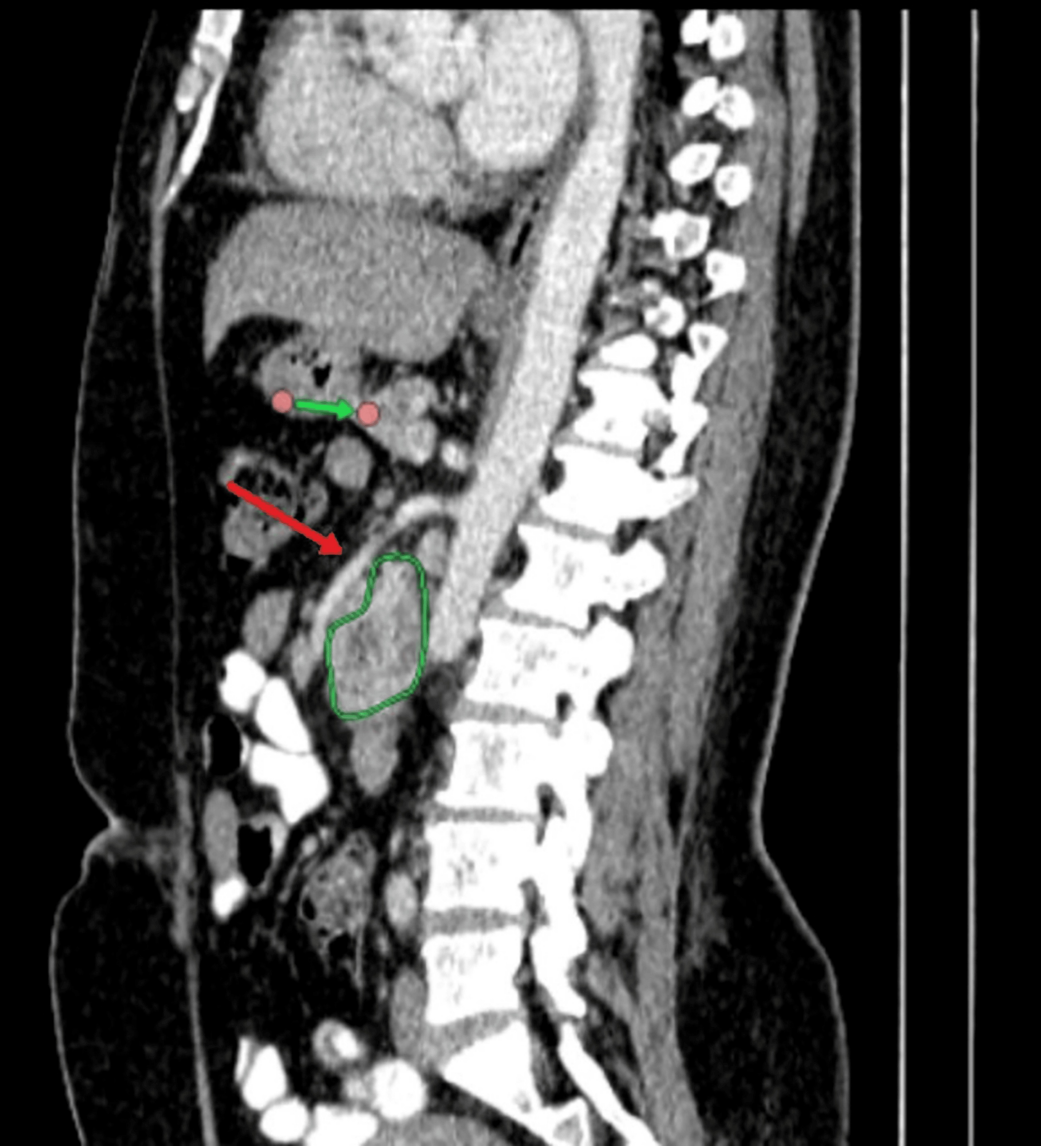
Sagittal view of the abdomen CT scan shows a well-defined mass in the duodenum (outlined in green) with the red arrow indicating its proximity to nearby vascular structures. This mass is consistent with a GIST. GIST: Gastrointestinal stromal tumor

Pelvi-abdominal US showed a large epigastric lesion measuring 65 mm x 45 mm with central cavitation, likely originating from the bowel or pancreas. The liver and spleen were normal in size and texture, with no visible lymph nodes or ascites. The CT abdomen with contrast showed a retroperitoneal, well-defined, lobulated soft tissue lesion, centered on the third and fourth parts of the duodenum, measuring 4.6 cm x 8.4 cm x 5.8 cm (Figures 1-2). The lesion exhibited heterogeneous, predominantly peripheral post-contrast enhancement with central hypodense necrotic areas. It was abutting the undersurface of the pancreatic uncinate process, encasing the proximal branches of the superior mesenteric artery and vein, and abutting the ventral surface of the infrarenal segment of the inferior vena cava (IVC) and the aorta.

The patient was transferred to the surgery department shortly after her initial evaluation. During the surgery, the lesion was confirmed to be of duodenal origin with significant vessel encroachment. An intraoperative decision was made to proceed with a pancreaticoduodenectomy (Whipple procedure). The resected lesion was sent for pathological analysis.

Pathological examination of the resected duodenal mass revealed a submucosal neoplasm composed of CD117-positive spindle cells with many dilated vascular spaces, wide areas of hemorrhage, and >10 mitotic count per 50 high-power field (HPF). The neoplasm was confined to the submucosa and muscularis propria, with focal involvement of the mucosa but without invasion of the serosa, confirming the diagnosis of a high-risk D-GIST. The surgical margins were clear of tumor involvement, and the dissected lymph nodes showed reactive follicular hyperplasia without evidence of tumor spread.

By the time of discharge, the patient's HB had risen to 10 g/dl. She was discharged after a period of observation, with follow-up scheduled one week later to assess for perioperative complications. A follow-up schedule for possible recurrence was also put in place, with the next appointment scheduled four months after the surgery.

## Discussion

Duodenal GISTs are rare mesenchymal tumors originating from the interstitial cells of Cajal or precursor cells in the muscularis propria of the duodenum [1]. They are infrequent, accounting for a small fraction of all GIST cases [2]. They are less common compared to GISTs located in the stomach or small intestine [2]. They are typically diagnosed in older adults with a median age of approximately 60 years and no significant difference in incidence according to gender [3]. Being 48 years old, our patient had a relatively early onset of disease compared to the general population.

Duodenal GISTs often reach a diameter of 5 cm before they become symptomatic [4]. They grossly present as a subendothelial lesion (SEL), and on microscopic examination, they can display spindle cell, epithelioid, or pleomorphic patterns [5]. They may contain skeinoid fibers, especially in low mitotic rate cases [5]. High mitotic rates and variations in morphology, including large and sarcomatous forms, can influence prognosis [5]. They typically express CD117 (KIT) and sometimes CD34 [6]. In our case, CD117-positive cells with areas of hemorrhage and necrosis and a high mitotic index were reported, signifying high-risk D-GIST.

The presentation of GISTs is variable, with many being discovered incidentally and others presenting with abdominal pain, bleeding, or obstruction [3]. Diagnosis is often based on imaging studies and confirmed with biopsy and immunohistochemistry [6]. Imaging modalities include CT, MRI, and EUS [7]. Tumors larger than 5 cm and with high mitotic rates have a poorer prognosis [2]. Complications include local invasion, as large GISTs can invade surrounding structures, including the pancreas and ampulla of Vater, complicating surgical management [5]. Metastasis, although rare, can be present, most commonly to the liver or peritoneum [2].

Management varies based on tumor size and location [4]. Options include wedge resection, segmental resection, pancreatic head-preserving duodenectomy, and pancreaticoduodenectomy. Laparoscopic approaches are feasible and offer advantages in terms of minimally invasive treatment [4]. Tumor size is a significant predictor of recurrence-free survival (RFS) and overall survival (OS), being a more accurate predictor of survival than microscopic examination of margins of resection [6].

Both CT and MRI are essential for diagnosis, staging, and monitoring treatment response [7]. Each modality has specific advantages in evaluating GIST characteristics and response to therapy [7]. However, EUS is considered a key test for differential diagnosis of SELs [7-10]. It can show which gastrointestinal wall layer the tumor originates from, as well as its nature and size [7-10]. Therefore, EUS is considered the safest and most useful modality for differential diagnosis and follow-up of SELs [8-10]. It can help discriminate between GISTs and other SELs including lipomas, cysts, varices, and extraintestinal compression [8]. However, EUS imaging alone provides insufficient accuracy in diagnosing GISTs, so tissue sampling for immunohistochemical analysis using EUS-FNA or biopsy is required whenever possible for a definite diagnosis before surgery or chemotherapy [8,11-13]. For our case, we opted for surgery based on the findings of the EUS without taking a biopsy due to the high bleeding risk.

Regarding the utility of EUS in predicting malignancy risk, possible high-risk features for GISTs include a size of > 2 cm, irregular borders, heterogeneous echo patterns, anechoic spaces, echogenic foci, and growth during follow-up [14,15]. However, at present, estimation of the risk of malignancy of GISTs of < 5 cm by EUS imaging alone seems to be difficult [8]. A finding like a hypoechoic solid mass by EUS is seen in malignant and benign conditions alike, including malignant lymphoma and benign leiomyoma, respectively [8,16]. It is difficult to distinguish between these lesions using EUS findings only [8].

Due to previously mentioned limitations, tissue sampling for immunohistochemical analysis using EUS-FNA or biopsy is required for definite diagnosis and appropriate management [15,17,18]. The EUS is the preferred imaging modality used in the diagnosis of GIST and is the most established diagnostic method when combined with FNA and immunohistochemistry [19]. Typical EUS-FNA findings of GISTs are KIT- or CD34-positive spindle-shaped cells or epithelial cells [19]. It should also be noted that accuracy increases as tumor size increases. Therefore, EUS-FNA is recommended for masses of > 1 cm [19].

Regarding treatment, surgical resection is the first choice for resectable GISTs without metastasis [19,20]. For unresectable, metastatic, or recurrent GISTs, administration of tyrosine kinase inhibitors such as imatinib is done [19,20]. Lymph node dissection is not recommended except when lymph node metastasis is clinically suspected [20]. Other minimally invasive techniques with endoscopy can be attempted for smaller tumors [20].

The first and second lines of imatinib are used in the therapy for unresectable tumors, leading to improved mortality, and in high-risk resectable tumors as an adjuvant therapy to decrease recurrence and induce cure [21-26]. Sunitinib is usually given in cases of treatment failure with imatinib [21-23]. Additionally, for c-KIT-positive tumors, mutational analysis should be done to rule out resistance against imatinib [24-26].

However, determining the prognosis of cases of D-GIST remains difficult even with the above-mentioned measures. Currently, the modified Fletcher classification (Joensuu classification) is used for risk assessment (Table 3) [3,27,28]. In addition, contour maps can be formed using the diameter, number of mitoses, tumor size, and capsule status [29]. These maps will help in assessing the 10-year recurrence risk [29]. This is useful for individual decision-making with respect to adjuvant therapy, as stated above in the discussion of the role of imatinib [29]. The high mitotic index noted in our case makes it a high-risk GIST.

**Table 3:** Modified Fletcher’s risk classification Also called Joensuu classification [28], this is a modified classification based on the Fletcher classification [27].

Risk category	Tumor size (cm)	Mitotic index (per 50 HPFs)	Primary tumor site
Very low risk	< 2.0	≤ 5	Any
Low risk	2.1-5.0	≤ 5	Any
Intermediate risk	2.1-5.0	> 5	Gastric
	< 5	6-10	Any
	5.1-10.0	≤ 5	Gastric
High risk	Any	Any	Tumor rupture
	> 10 cm	Any	Any
	Any	> 10	Any
	> 5.0	> 5	Any
	2.1-5.0	> 5	Non-gastric
	5.1-10	≤ 5	Non-gastric

Postoperatively, follow-up using abdominal contrast CT is advised for early detection and management of recurrence [30]. Follow-up schedule varies according to assessed risk (Table 4). Few recurrences were reported after the first 10 years of follow-up [8,28,30]. As such, follow-up observation after surgery is considered necessary for at least 10 years [30].

**Table 4:** Follow-up schedule according to assessed risk GIST: Gastrointestinal stromal tumor

Risk classification	Follow-up schedule
Very low, low, and moderate risks	Follow-up CT every six months to one year
High-risk and clinically malignant GISTs	Follow-up CT every four to six months

## Conclusions

This case report underscores the critical role of comprehensive imaging in accurately diagnosing D-GISTs, particularly given their atypical presentation that can resemble other conditions like pancreatic masses. The successful resection of the D-GIST through a pancreaticoduodenectomy coupled with a definitive histopathological diagnosis emphasizes the role of multidisciplinary management in improving patient outcomes. Long-term follow-up is essential to monitor for potential recurrence, especially in high-risk GISTs, to ensure optimal long-term prognosis.
